# Can Physical Activity Support Grief Outcomes in Individuals Who Have Been Bereaved? A Systematic Review

**DOI:** 10.1186/s40798-021-00311-z

**Published:** 2021-04-08

**Authors:** Jane Williams, Gillian W. Shorter, Neil Howlett, Julia Zakrzewski-Fruer, Angel M. Chater

**Affiliations:** 1 Institute for Sport and Physical Activity Research, Centre for Health, Wellbeing and Behaviour Change, University of Bedfordshire, Bedford, MK41 9EA UK; 2 Centre for Improving Health Related Quality of Life, School of Psychology, Queen’s University Belfast, Belfast, BT9 7NN UK; 3 Department of Psychology and Sport Sciences, University of Hertfordshire, Hatfield, AL10 9AB UK

## Abstract

**Background:**

In 2018, there were 616,014 registered deaths in the United Kingdom (UK). Grief is a natural consequence. Many mental health concerns, which can be identified as grief outcomes (e.g. anxiety and depression) in those who have experienced a bereavement, can be improved through physical activity. The objective of this review was to identify from the existing literature if physical activity can benefit grief outcomes in individuals who have been bereaved.

**Methods:**

A systematic review of nine databases was performed. Included studies (qualitative and quantitative) explored physical activity to help individuals (of any age) who had experienced a human bereavement (excluding national loss).

**Results:**

From 1299 studies screened, 25 met the inclusion criteria, detailing eight types of bereavement (parental (*n* = 5), spousal (*n* = 6), patient (*n* = 4), pre-natal (*n* = 3), later life (*n* = 1), caregiver (*n* = 1), multiple (*n* = 4) and non-defined (*n* = 1). Activities including yoga, running, walking and martial arts were noted as beneficial. Physical activity allowed a sense of freedom, to express emotions, provided a distraction and an escape from grief, whilst enhancing social support.

**Conclusion:**

There is some evidence that physical activity may provide benefit for the physical health and psychological wellbeing of those who have been bereaved, including when the loss has happened at a young age. This review is timely, given the wide-scale national loss of life due to COVID-19 and extends knowledge in this area. More research is needed to explore the benefits of physical activity for those who have been bereaved. In particular, there is a need for well-designed interventions which are tailored to specific activities, populations and grief outcomes.

## Key Points

- Many grief outcomes following a bereavement manifest in physical and mental health concerns. Physical activity is known to have a significant positive impact on physical and mental health, yet its impact on these factors, that could be as a result of grief outcomes for those who have been bereaved, is unknown.
- This review suggests that physical activity may benefit grief outcomes in individuals who have been bereaved, alleviating feelings of depression, anxiety and the experience of post-traumatic stress disorder; whilst also creating a sense of freedom, enabling the expression of emotions, providing a distraction, and an escape from grief.
- This is the first review of its kind, which highlights the possible benefits of physical activity to those who have been bereaved. Given the impact bereavement can have, further research in this area is warranted.

## Background

There were 616,014 registered deaths during 2018 in the UK alone [1–3]. Bereavement is a common experience following death [4] and is a term often used interchangeably with grief and mourning; yet they differ in meaning. Following a bereavement, individuals grieve and mourn in different ways. Bereavement refers to being in a state of loss as the result of the death of a significant relationship, mourning is an expression of grief and grief is the complex bio-psycho-social response and reaction to bereavement [5, 6]. Individuals may experience a range of grief outcomes following a bereavement such as increased levels of anxiety and depression [5], self-harm [6], alcohol and drug use [7] and suicide ideation or attempts [8]. Individuals may experience a decrease in concentration [9] and self-esteem [10], or they may experience insomnia, aggression [11], or post-traumatic stress [5]. Individuals can experience multiple grief outcomes simultaneously, at different rates, intensities and durations [12]. There are a multitude of factors which can influence how a person grieves; these include their age, the type of death and their relationship to the deceased [5, 13]. Children and young people have a different understanding of death to adults, and they may not fully understand the situation or their feelings relating to it [5, 9, 12]. Experiencing a bereavement as a result of murder may leave individuals with increased aggression, guilt, unanswered questions and traumatic imagery [14, 15]. This type of death can additionally delay the grieving process as the family may not be able to grieve during the criminal justice trial [16]. Some bereavements, for example those from death by suicide, may also lead to prolonged grief disorder (PGD), a term used to describe an ongoing, heightened sense of mourning and rumination [17], leaving individuals with an increased sense of guilt [18]. PGD is categorised as grief outcomes which fail to diminish after 6 months causing disruption to daily, social and occupational functioning [17, 19]. Experiencing a traumatic bereavement such as death by suicide as a child or young person can increase suicidal ideation and attempts when compared to those who experienced bereavement due to a natural death [20, 21].

Those experiencing grief outcomes from both natural and sudden or traumatic bereavements should be able to seek appropriate support. Within the UK, there are nationwide and local regional bereavement services catering to all ages. Services such as Cruse, Child Bereavement UK, Hope Again and Winston’s Wish have centres around the UK, with CHUMS, Simon Says, Halo and several more offering support in specific localities. Each of these services offer a variety of bereavement support, including individual or group counselling, telephone support, activity days and weekend retreats.

As many grief outcomes such as depression, anxiety, anger, lowered self-esteem, substance use, self-harm and suicide ideation are also mental health concerns, without the experience of a bereavement, it is plausible to suggest that options to improve mental health may well improve these issues when manifested as grief outcomes. One way to improve mental health is through physical activity; and a number of studies have found that it can benefit and reduce factors such as depression [22–24] and anxiety [25–27]. Physical activity has been shown to reduce aggression [28, 29], improve life satisfaction [30] and reduce post-traumatic stress disorder (PTSD) [31]. Adventurous physical activity (e.g. rock climbing) improves positive and negative affect and self-efficacy [32]. However, the extent to which physical activity may be beneficial to such outcomes, in those who have been bereaved, is unknown.

This review aims to identify studies which have investigated the role of physical activity following a bereavement, with a specific question as to whether physical activity can benefit grief outcomes. It further seeks to understand what grief outcomes are commonly recorded, how they are measured and the types of physical activities reported to be helpful to those who have experienced a bereavement.

## Methods

### Protocol Registration

This review is reported using the Preferred Reporting Items for Systematic Reviews and Meta-Analyses (PRISMA) guidelines. A protocol for this review has been registered with PROSPERO (Ref: CRD42017081237).

### Eligibility Criteria

Eligible studies were not restricted by year, nor by study design, but were restricted to those written in the English language. Study characteristics were defined using PICOS (Population, Intervention, Comparator, Outcome, Study design [33]) as below to identify relevant sources.

### P—Population

Population were of any age and sex and must have experienced personal grief (e.g. death of someone they knew). Studies that described those who had experienced national grief (e.g. ‘9/11’ or death of a monarch) were excluded. Populations grieving the loss of a pet, activity or a sporting injury (e.g. loss due to retiring from sports) were also excluded.

### I—Intervention

Studies were included if physical activity was used to support bereavement, grief or mourning, or if physical activity was included as a measurement in relation to bereavement. Physical activity was defined as any activity that uses the skeletal muscles and requires an energy expenditure of above 1.5 metabolic equivalent (MET), which is considered resting [34]. Physical activity could range from light to vigorous intensity and be of any duration or mode (e.g. walking, football, dance). Studies which used physical activity with the primary aim to prolong life, reduce premature death, or linked to mortality were excluded.

### C— Comparison

This review did not restrict inclusion by comparator.

### O—Outcomes

The primary outcomes were cognitive, affective and behavioural grief outcomes. Cognitive/affective grief outcomes included anxiety, depression, anger, guilt, loneliness, post-traumatic stress, self-esteem, suicidal ideation, well-being, resilience, life satisfaction and quality of life. Behavioural grief outcomes included alcohol or drug use, emotional eating, isolation, insomnia, self-harm and suicide attempts. Other outcomes not listed but that were attributed as grief outcomes were documented.

### S—Study Design

All study designs were included in this review.

### Information Sources

This systematic review was performed during May 2019 and updated in November 2020 and includes studies from the inception of the databases. Initially, the Cochrane Library and PROSPERO were searched for relevant reviews to avoid duplication. A comprehensive search of the following electronic databases was then performed: BASE, Directory of Open Access Journals, Medline PubMed, PsycArticles, PsycINFO, Science Direct, Scopus and SPORTDiscus. Once all appropriate articles were identified, cited references were hand searched for further appropriate articles.

### Search Strategy

A combination of terms from medical subject headings (MeSH) and keyword variants were used to identify records. Search terms found in Table 1 were used and adapted to each database search.

**Table 1:** Eligibility criteria based on PICOS study characteristics and search terms

Concept	Search terms
Population	MeSH Terms: Bereavement, grief
	Free text terms: Parental death, spousal death, sibling death, family death, child death, stillbirth
Intervention	MeSH Terms: Exercise, sport
	Free text terms: physical activit*, sport, exercise, outdoor physical activity, adventure therapy
Comparison	None specified
Outcomes	MeSH Terms: Anxiety, depression, guilt, loneliness, suicide, substance related disorders, insomnia
	Free text terms: anxiety, depress*, guilt*, anger, loneliness, post-traumatic stress, self-esteem, suicid*, well-being, resilience, substance *use, substance related disorder, emotional eating, isolation, insomnia, self-harm, concentration, quality of life, life satisfaction
Study Design	None specified

### Study selection

Records were imported into Mendeley [35] software with any duplicates removed by JW. All titles and abstracts were screened for clear violation of inclusion criteria followed by 100% full text screening for eligibility independently by JW and GWS. Any discrepancies in decision making were discussed and resolved with AMC.

### Data Collection Process

Two reviewers (JW, AMC) independently extracted and inserted data from 100% of the articles into Excel, using a predefined data extraction form. This form was piloted and updated to include additional extraction categories for clarity using two initial studies. The final version included study title, authors, journal, publication date, country of study, study design characteristics (including type of study and any comparator groups), recruitment method and funding source. Participant characteristics included sample size, demographics (age, sex, ethnicity, religion), relationship to deceased, length of and mean time since the bereavement. Interventions were described using the TIDieR (Template for Intervention Description and Replication) checklist and guidance framework [36], where relevant information such as intervention fidelity, tailoring, adaptions, provider, number of sessions, procedure, delivery, materials, type and length of the intervention were recorded. The type of physical activity, measures used, theoretical frameworks, follow-up periods and any behaviour change techniques [37] were recorded. It was noted if a control group was used. Outcome data recorded the aim, main outcomes, grief outcomes and measurements used.

### Risk of Bias in Individual Studies

Two reviewers (JW, GWS) independently assessed the quality of the eligible studies using the Mixed Methods Appraisal Tool (MMAT) [38]. The MMAT supports the assessment of the quality of qualitative, quantitative (randomised, non-randomised and descriptive) and mixed methods studies. The quality of each study was ranked from 25 to 100% by answering ‘yes’, ‘no’ or ‘can’t tell’ to a series of questions. The MMAT questions centre around the relevance of study design (*Are there clear qualitative and quantitative research questions* (*or objectives*), *or a clear mixed methods question* (*or objective*?), sources (*Are the sources of qualitative data* (*archives*, *documents*, *informants*, *observations*) *relevant to address the research question* (*objective*?), participant randomisation (*Are participants* (*organisations*) *recruited in a way that minimizes selection bias*?), appropriate measures (*Are measurements appropriate* (*clear origin*, *or validity known*, *or standard instrument*)*?*), response rate (*Is there an acceptable response rate* (*60*% *or above*?), comparison groups, complete outcome data (*Are there complete outcome data* (*80*% *or above*) and, when applicable, an acceptable response rate (*60*% *or above*), or an acceptable follow-up rate for cohort studies (*depending on the duration of follow*-*up*?), participant blinding (*Is there a clear description of the allocation concealment* (*or blinding when applicable*?) and drop-out rates (*Is there low withdrawal*/*drop*-*out* (*below 20*%?).

### Synthesis of Results

The impact of physical activity with any available measurement of the primary grief outcomes was reported. A meta-analysis was not possible due to the variety of outcomes and study designs; therefore, a narrative synthesis was performed.

## Results

### Study Selection

**Fig. 1:**
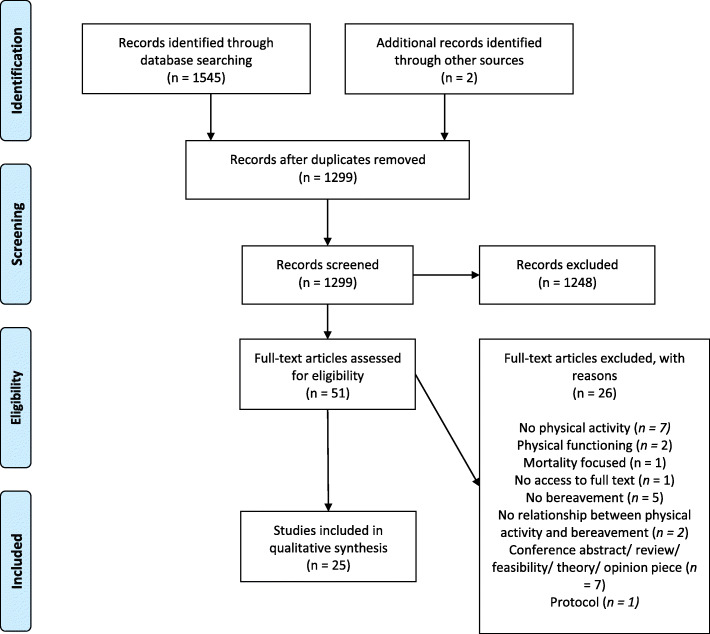
PRISMA diagram for the systematic review of the role of physical activity following a bereavement

A total of 1545 titles and abstracts were initially identified from the search criteria, with an additional two included from hand searching. Of these, 248 duplicates were removed, leaving 1299 records to be screened. Of these, 1248 titles and abstracts were excluded, leaving 51 articles for full text review. A total of 25 of these articles met the inclusion criteria and were included in the analysis (see Fig. 1).

### Study Characteristics

Within these articles, nine of the studies used qualitative interviews and observations, 14 were quantitative using a variety of questionnaires as measurement tools; two of the studies used mixed methods of qualitative and quantitative methods. Of the 14 quantitative studies, seven of these were experimental designs [39–45]. The eligible articles were from eleven different countries, the majority in the USA (*n* = 10) [39–41, 44, 46–51], followed by the UK (*n* = 4) [52–55], Canada (*n* = 2) [56, 57], South Korea (*n* = 1) [45], Israel (*n* = 1) [58], China (*n* = 1) [59],Sweden (*n* = 1) [60], Africa (*n* = 1) [61] and Germany (*n* = 1) [62], Hong Kong (*n* = 1) [43] and Korea (*n* = 1) [42]. There was one study with an unknown location [63]. An overview of the study characteristics and findings can be found in Table 2.

**Table 2:** Summary of data extraction to understand the role of physical activity in individuals who have been bereaved

Author (year) and country [Ref Number]	Title of article	Study type (Methods)	Sample size	Participant details Age range (Mean) Sex	Type of bereavement [length of bereavement (mean)]	Grief outcome (measure used)	Type of physical activity	Main findings
Brewer and Sparkes (2011) UK [53]	Young people living with parental bereavement: Insights from an ethnographic study of a UK childhood bereavement service	Qualitative (ethnography—interviews and observations)	13	9–25 years (25.15) Male (*n* = 6) Female (*n* = 7)	Parental death [2–15 years (9.75)]	Aggression, anxiety, depression.	Martial arts, football, walking, running	Seven key themes related to a bereavement camp experience identified: (1) expressing emotion, (2) physical activity, (3) positive adult relationships, (4) area of competence, (5) friendships/social support, (6) having fun/humour and (7) transcendence
Brewer and Sparkes (2011) UK [54]	The meanings of outdoor physical activity for parentally bereaved young people in the United Kingdom: insights from an ethnographic study	Qualitative (semi-structured interviews and observations)	13	9–25 years (25.15) Male (*n* = 6) Female (*n* = 7)	Parental death [2–15 years (9.75)]	Anxiety, aggression, panic attacks, stress	Martial arts, rugby, sports, exercise	Physical activity provided: (1) a sense of freedom, (2) a distraction/escapism, 3) enabled memories to be retained and 4) created family cohesion
Chen et al. (2005) USA [41]	Health behaviours associated with better quality of life for older bereaved persons	Quantitative (questionnaire)	200	50+ years (66.3) Male (*N* = 53) Female (*N* = 147)	Later Life bereavement [no range or mean]	Quality of life, depression, stress (RAND-36)	Exercise (Not Specified)	Exercising one or more days per week consistently predicted better quality of life outcomes.
Gorman and Cacciatore (2020) USA [46]	Care-farming as a catalyst for healthy and sustainable lifestyle choices in those affected by traumatic grief	Qualitative (questionnaire and semi-structured interviews)	120	18–55 years Male (*n* = 21) Female (*n* = 99)	Multiple bereavements [no range or mean]	Grief [non-defined]	Walking, Kayaks, non-defined physical activity	Bereaved parents, siblings and spouses described significant pivots toward healthier eating, sleeping and increased physical activity. Care-farming may have a potential influence in positive changes to health and health behaviours. Experiences at the care-farm prompted an uptake in physical activity in the outdoors.
Granek et al. (2017) Canada [56]	Experiences of Canadian oncologists with difficult patient deaths and coping strategies used	Quantitative (online questionnaire)	98	20–61+ (No mean) Male (*N* = 50) Female (*N* = 48)	Patient death [0–3+ deaths per month (no mean)]	Coping strategies	Exercise (not specified)	A broad variety of coping strategies were used in responding to patient death. Exercising and *watching* sports were among those used as a coping strategy.
Granek et al. (2016) Israel [58]	Barriers and facilitators in coping with patient death in clinical oncology	Qualitative (interviews—grounded theory)	22	32–70 years (47) Male (*N* = 14) Female (*N* = 8)	Patient death [1–25 deaths per month (5)]	Coping strategies	Watching soccer, sports (not defined)	Coping strategies for a patient death included cognitive, behavioural, relational, professional and spiritual methods. Participating or watching sports were included within behavioural coping strategies.
Granek et al. (2016) Canada [57]	Paediatric oncologists' coping strategies for dealing with patient death	Qualitative (interviews—grounded theory)	21	31-74 years (43) Male (*N* = 10) Female (N=11)	Patient death [1–13 deaths per month (1.5)]	Coping strategies	Walking, yoga, running, cycling, hiking, dancing	After a patient’s death, physical activity was an important coping strategy; it allowed oncologists to ‘let off steam’. Oncologists participated in a variety of regular physical activities, often outdoors and in nature.
Grimby et al. (2008) Sweden [60]	Walking habits in elderly widows	Mixed methods (questionnaire and interviews)	701	51–89 years (76)	Spousal death [3 months–4 years (no mean)]	Stress	Walking	Initially following the death of a spouse, there was a reduction in physical activity, with women having a reduced perception of being healthy. This physical activity, and perception increased with time after bereavement.
Gyasi and Phillips (2019) Africa [61]	Risk of psychological distress among community-dwelling older adults experiencing spousal loss in Ghana	Mixed methods (interviews and questionnaires)	1200	50+ Male (*N* = 759) Female (*N* = 441)	Spousal death [no range of mean]	Psychological distress	Non-defined sports	Psychological distress, increased with spousal loss, in women but not in men. The association between spousal loss and psychological distress was reduced as social support resources and physical activity was increased.
Huberty et al. (2014) USA [47]	A qualitative study exploring women's beliefs about physical activity after stillbirth	Qualitative (interviews)	24	19–44 years (33) Female (*N* = 24)	Foetal/pre-natal death [< 12 months (6.33)]	Depression, quality of life	Physical activity	The major barriers to physical activity were emotional responses, lack of motivation, feeling tired, guilt, letting go of the pregnancy body, seeing other babies and lack of time. Physical activity allowed them to feel better emotionally, helping them to cope; gave time for themselves to work through grief and was motivated by body shape/weight.
Huberty et al. (2014) USA [48]	Physical activity and depressive symptoms after stillbirth: Informing future interventions	Quantitative (descriptive exploratory)	175	19–45 years (31.26) Female (*N* = 175)	Foetal/pre-natal death [0–12 months (no mean)]	Depression (EPDS)	Yoga, walking, jogging	38% of women used physical activity to cope with depression following a stillbirth. Women participated in the recommended guidelines for physical activity: 60% before stillbirth, 47% during pregnancy and 61% after still birth. Of those that reported using physical activity to cope after stillbirth, they did so to help with depression (58%), weight loss (55%), and better overall physical health (52%). To cope with stillbirth, women used walking (67%), jogging (35%), and yoga (23%).
Huberty, et al., (2020) USA [40]	Online yoga to reduce post-traumatic stress in women who have experienced stillbirth: a randomised controlled feasibility trial	Quantitative (RCT)	90	18+ Female (*N* = 90)	Foetal/pre-natal death [6 weeks–24 months (40.92 weeks)]	PTSD, depression, anxiety, emotional regulation	Yoga, stretch and tone	There were significant decreases in PTSD and depression; and improvements in self-rated health, post intervention for those within the intervention (yoga) conditions.
Kang and Yoo (2007) Korea [42]	Effects of a bereavement intervention program in middle-aged widows in Korea	Qualitative (quasi-experimental)	27	36–64 (intervention 55.8, control 54.4) Female (*N* = 27)	Spousal death [2–6 months (no mean)]	Grief, stress, immune response	Dan-Jeon breathing and stretching	An experimental group, participating in Dan-Jeon breathing sessions, a self-help group activity, and a health check showed significantly greater decrease in grief levels and symptoms of stress compared to a control group who received only a health check.
Li et al., (2015) Hong Kong [43]	From body to mind and spirit: qigong exercise for bereaved persons with chronic fatigue syndrome-like illness	Quantitative (intervention)	46	23–52 years (intervention 46 [median], control 45 [median]) Male (*N* = 6) Female (*N* = 40)	Spousal death, sibling death, parental death, other death [< 2 years (no mean)]	Fatigue, depression, anxiety, well-being	Qigong	Bereaved participants with an illness likened to chronic fatigue syndrome had significantly higher mental fatigue scores and lower physical functioning than non-bereaved participants. Participants within the Qigoing intervention group had a significant decrease in mental and physical fatigue after 3 months compared to the control group.
McClatchey et al. (2009) USA [44]	Efficacy of a camp-based intervention for childhood traumatic grief	Quantitative (intervention)	100	6–16 years (no mean) Male (*N* = 48) Female (*N* = 52)	Parental death [0–48 months (Camp A 12.57 months), Camp B 15.11 months)	PTSD [DSM-IV] Childhood traumatic grief (Extended Grief Inventory)	Canoeing, hiking, treasure hunts, and other play activities.	A short term, trauma-focused grief camp reduced traumatic grief and post traumatic grief disorder in children who had been parentally bereaved. Children participated in a range of traditional camp activities (canoeing) and counselling. Symptoms of both traumatic grief and PTSD continued to decline at a 2-week follow-up.
McClatchey al. (2012) USA [49]	Healing components of a bereavement camp: Children and adolescents give voice to their experiences	Qualitative (semi-structured interviews)	32	8–17 years (11.47) Male (*N* = 5) Female (*N* = 27)	Parental death [not mentioned (no mean)]	Grief	Traditional camp activities: canoeing	Traditional camp activities such as canoeing were viewed as the most enjoyable and a healing element where children were able to connect with each other. Counselling was viewed as the most beneficial element of the grief camp.
Moores et al. (2007) UK [52]	Memorable patient deaths: Reactions of hospital doctors and their need for support	Quantitative (cross-Sectional)	188	N/A (no mean) Male (*N* = 100) Female (*N* = 85) Missing (*N* = 3)	Patient death [not mentioned (no mean)]	Coping strategies, appetite changes, fatigue, sleep, crying, numbness, emptiness, sadness	Exercise (not specified)	The most frequent coping strategy after experiencing patient death was talking with others (83.5%). Other coping mechanisms were: having time alone (64.4%), socialising (36.2%), exercise (26.2%) and religious guidance (21.8%).
Phoenix and Orr (2017) UK [55]	Analysing exceptions within qualitative data: promoting analytical diversity to advance knowledge of ageing and physical activity	Qualitative (narrative constructionism—interviews)	51	N/A (no mean) Male (*N* = 23) Female (*N* = 28)	General bereavement [not mentioned (no mean)]	Anxiety, depression	Physical activity (not specified)	Following the death of a loved one, physical activity was found to strengthen or generate new social networks. Results show that pre-arranged physical activity provided motivation, and a reason to get out of bed and interact with other individuals in a social environment.
Richardson (2010) USA [50]	Length of caregiving and well-being among older widowers: Implications for the dual process model of bereavement	Quantitative (survey)	200	58–91 years (75) Male (*N* = 200)	Spousal death [13–24 months (520 days)]	Affect	Social clubs, playing sports	Negative affect was influenced by time since death, ethnicity, and participation in clubs. Following the death of a spouse (wife), participants joined clubs and participated in sports often. Factors which influenced positive affect included length of caregiving, number of friends, and having a confidante.
Simpson et al. (2014) USA [51]	The impact of mid- and late-life loss on insomnia: Findings from the Health and Retirement Study, 2010 cohort	Quantitative (database—Health and Retirement Study: HRS)	12,759	50–70 years (no mean) Male (*N* = 6084) Female (*N* = 6675)	Spousal death, parental death, child loss, sibling [not mentioned (no mean)	Insomnia, depression	Active (not specified)	Those experiencing one or more bereavements in mid-later life were found to have significantly higher proportions of subclinical and clinical symptoms of insomnia than those with no loss even when age, sex, and lifestyle behaviours (smoking, alcohol use, BMI, and physical activity) were taken into consideration.
Stahl et al., (2020) USA [39]	Digital monitoring of sleep, meals, and physical activity for reducing depression in older spousally-bereaved adults: a pilot randomised controlled trial	Quantitative (RCT)	57	60+ (75) Males and females (no breakdown given)	Spousal death [< 8 months no mean)]	Depressive symptoms, anxiety, PTSD, suicidality	Physical activity (non-defined)	Behavioural interventions, that incorporate digital monitoring behaviours (sleep, diet, physical activity) and a motivational health coach, are feasible and acceptable to older bereaved adults at high risk of depression. Depression symptoms decreased pre to post intervention.
Wicker and Orlowski (2020) Germany [62]	Coping with adversity: physical activity as a moderator in adaption to bereavement	Quantitative (dataset—German socio-economic)	139,097	20–105 (51.21) Males (*N* = 66,352) Females (*N* = 72,745)	Multiple deaths [< 12 months (no mean)]	Life satisfaction, Subjective well-being	Physical activity (non-defined)	The results found that those individuals who were physically active in the past were found to adapt quicker to adverse life events, thus supporting the moderating effect of physical activity following bereavement.
Yoo and Kang (2006) South Korea [45]	Effects of a bereavement intervention program on depression and life satisfaction in middle aged widows in Korea	Quantitative (quasi-experimental design)	27	35–64 years (intervention 55.8, control 54.4) Female (*N* = 27)	Spousal death [0–6 months (no mean)]	Depression, life satisfaction	Dan-Jeon breathing and stretching	Participants within the experimental group, engaging in physical activity significantly decreased levels of depression when compared to the control group who had no physical activity engagement. In addition, participating in physical activity significantly increased life satisfaction.
Zhang et al. (2008) Country not given [63]	Depressive symptom trajectories and associated risks among bereaved Alzheimer disease caregivers	Quantitative (questionnaires)	182	(62.8) Male (*N* = 30) Female (*N* = 152)	Caregiver death [post loss assessment, 13.9,37.9, 63.6-week medians]	Depression	Exercise (not specified) caregiver scale—exercise for > 15–30 minutes at least 3 times per week)	Three depressive symptom trajectories reported: syndromal, syndromal-beginning-threshold and persistently absent depression. Risks of syndromal-beginning-threshold level depression were: lack of family support, caregiver burden and adverse health behaviours (e.g. physical inactivity). Having access to early intervention for these factors may decrease the risk of depression after loss.
Zhao et al. (2014) China [59]	Extracurricular interest as a resilience building block for children affected by parental HIV/AIDS	Quantitative (cross-sectional)	1625	6–18 years (12.25) Male (*N* = 826) Female (*N* = 799)	Parental death [not mentioned (no mean)]	Depression, loneliness, self-esteem	Sport (not specified)	Participating in extra-curricular activities including sports decreased the negative effects of parental death from HIV/AIDS in children. After controlling for age, gender, family and socioeconomic status, children’s self-esteem increased, and loneliness decreased by participating in extracurricular activities such as sport.

### Participant Characteristics

A total of 157,068 individuals participated across the 25 articles, with an age range of 6–91 years old. Studies included 47.5% males and 52% females; 0.5% could not be attributed as two papers failed to provide details on participant sex. The high overall sample size is due to two studies with a total of 139,097 [62] and 12,759 [51] participants respectively. Four ethnicities were reported across 12 studies, specifically Hispanic, Caucasian, Asian and African-American. Thirteen studies did not mention ethnicity [42, 43, 45–47, 52, 55–62] or just stated ‘other’ [48].

### Grief Outcomes

A total of 26 different grief outcomes were mentioned. Depression was most commonly mentioned in 12 studies [39–41, 43, 45, 47, 48, 51, 54, 55, 59, 63], followed by anxiety (*n* = 6) [39, 40, 43, 53–55], stress (*n* = 4) [41, 42, 54, 60], non-defined grief (*n* = 3) [42, 46, 49], PTSD (*n* = 3) [39, 40, 44], sleep problems/insomnia (*n* = 2) [51, 52], aggression (*n* = 2) [53, 54], life satisfaction (*n* = 2) [45, 62], quality of life (*n* = 2) [41, 47], fatigue (*n* = 2), well-being (*n* = 2) [43, 62], immune responses (*n* = 1) [42], general affect (*n* = 1) [50], childhood traumatic grief (*n* = 1) [44], self-esteem (*n* = 1) [59], panic attacks (*n* = 1) [54], loneliness (*n* = 1) [59], crying (*n* = 1) [52], emptiness (*n* = 1) [52], sadness (*n* = 1) [52], numbness (*n* = 1) [52], change in appetite (*n* = 1) [52], suicidal ideation (*n* = 1) [39], psychological distress (*n* = 1) [61] and emotional regulation (*n* = 1) [40]. Whilst not defined as grief outcomes, developing coping strategies were mentioned in four studies [52, 56–58].

### Types of bereavement

Eight different types of bereavement were reported within the included studies: spousal bereavement (*n* = 6), parental bereavement (*n* = 5), health professional-to-patient bereavement (*n* = 4), multiple bereavement (*n* = 4), bereavement following still-birth (*n* = 3), later-life bereavement (*n* = 1), caregiver bereavement (*n* = 1) and non-specified bereavement (*n* = 1).

### Length of Bereavement

The time since death related to the bereavement varied between studies, from 0 months to 15 years. Length of time since death was omitted from six studies [41, 46, 49, 51, 52, 55, 59, 61]. The longest length of time since death was 15 years [53, 54]. Eleven studies examined recent bereavement up to two years since death. Length of time participants had known the person who died was not routinely reported.

### Types of Physical Activity

There were 22 different physical activities mentioned. Brewer and Sparkes [53, 54], through interviews, found activities such as martial arts, rugby, football, running and walking were beneficial following parental bereavement. Others [44] found canoeing, hiking, treasure hunts, and other play activities helped with PTSD in young people who had been bereaved of a parent. These findings were supported [49], with traditional camping activities such as canoeing being found to be helpful after parental bereavement. Dan-Jeon breathing and stretching was found to decrease levels of stress and increase life satisfaction [42, 45]. Qigong (similar to Tai Chi) was found to reduce feelings of fatigue [43]. Physical activities such as yoga, walking and running were reported [40, 47, 48] to be useful in supporting grief following pre-natal death (still-birth). Walking was also found to be supportive for widowed individuals following the death of a spouse [60].

The above studies provided details of the type of physical activity used or mentioned within the research as it was a main aim or focus. Others without such focus found activities including sports, spending time outdoors, cycling, hiking, yoga and running were effective coping strategies used by medical practitioners after the death of a patient [56–58]. A number of studies failed to record type of physical activity when investigating its impact on grief outcomes. These were often studies which used cross-sectional designs, accessed large databases or used health behaviour questionnaires that were asking about generic physical activity performance [39, 41, 46, 47, 50–52, 55, 56, 58, 59, 61, 63].

### Behavioural Change Techniques Used in Interventions

Commonly, eligible papers did not provide enough detail on techniques related to increasing physical activity. Two studies [42, 45] report on the same project whereby intervention participants were given a demonstration led by a Dan-Jeon master, weekly instructions on performance, and the opportunity to practice stretching activities. Using the behaviour change technique (BCT) Taxonomy Version 1 [64], these studies used BCTs: 6.1 (Demonstration of the behaviour), 9.1 (Credible source), 4.1 (Instruction on how to perform the behaviour) and 8.1 (Behavioural practice/ rehearsal). Another study [39] used the same four BCTs, with the additional BCT 2.3 (Self-monitoring of behaviour). Within this study, participants were provided with a demonstration, instructed for the first 5 weeks, practiced stretching exercises which were led by a Qigong master and self-monitored their physical activity. One study [39] focused on digital monitoring of health behaviours, using BCTs 2.2. (Feedback on behaviour) and 2.1 (Monitoring of behaviour without feedback). Another study of an online yoga programme [40] used BCT 9.1 (Credible source), utilising a reliable source for yoga videos. BCT 12.5 (Adding objects to the environment) and 4.4 (Instructions on how to perform behaviour) were also used within the intervention, by providing yoga equipment, and instructions on how to use the videos. BCTs were unable to be coded for all other studies, often as they were not interventional in nature.

### Risk of Bias in Individual Studies

From the eligible studies, using the Mixed Methods Appraisal Tool [36] to assess study quality (see Table 3), ten articles were rated with 100% [44, 49, 52–55, 57–59, 62], 11 scored 75% [39–43, 45, 47, 48, 50, 60, 63], with the remaining four scoring 50% [46, 51, 56, 61].

**Table 3:** Mixed methods appraisal tool (MMAT) scores

Author (year)	MMAT Score				
	0%	25%	50%	75%	100%
Brewer and Sparkes (2011) [7, 53, 54]					X
Brewer and Sparkes (2011) [7, 53, 54]					X
Chen et al. (2005) [41]				X	
Gorman and Cacciatoire (2020) [46]			X		
Granek et al. (2017) [56]			X		
Granek et al. (2016) [57, 58]					X
Granek et al. (2016) [57, 58]					X
Grimby et al. (2008) [60]				X	
Gyasi and Phillips (2019) [61]			X		
Huberty et al. (2014) [47]				X	
Huberty et al. (2014) [48]				X	
Huberty et al. (2020) [40]				X	
Kang and Yoo (2007) [42]				X	
Li et al. (2015) [43]				X	
McClatchey et al. (2009) [44]					X
McClatchey and Wimmer (2012) [49]					X
Moores et al. (2007) [52]					X
Phoenix and Orr (2017) [55]					X
Richardson (2010) [50]				X	
Simpson et al. (2014) [51]			X		
Stahl et al. (2020) [39]				X	
Wicker and Orlowski (2020) [62]					X
Yoo and Kang (2006) [45]				X	
Zhang et al. (2008) [63]				X	
Zhao et al. (2014) [59]					X

### Summary of Studies

A synthesised narrative of the overall results provides evidence which suggests that physical activity can support grief outcomes following a bereavement. Physical activity was found to allow those who have experienced a bereavement to express emotions, escape from grief, retain memories and gain a sense of freedom [53, 54]. Participating in physical activity allowed individuals to create friendships, drawing upon social support, whilst also creating closer family cohesion [49, 54]. Physical activity enabled individuals who have experienced bereavement to reduce levels of depression, stress, loneliness and PTSD [42, 44, 48, 59, 63]. Medical professionals used sport and physical activity as a coping strategy after experiencing patient death [52, 56–58]. Engagement in physical activity was, however, reported to reduce following a bereavement [60], with some identifying barriers such as guilt, lack of time, fatigue and no motivation to be active, for this reduction [47]. However, it was acknowledged that physical activity levels increased with time [60] and that once barriers were overcome, it helped individuals to feel better following a bereavement [48].

## Discussion

This is the first review of its kind that suggests that physical activity may be beneficial for individuals following a bereavement, with some evidence that it can benefit several grief outcomes. This was found across different types of bereavement including parental, spousal, foetal and patient. Whilst there was general agreement between the studies that physical activity provided some benefit following bereavement, this benefit could not be matched to one single grief outcome. Depression was the most commonly mentioned grief outcome, and others most often fell within the ‘mental health’ umbrella (e.g. anxiety, stress, guilt, negative affect). This supports previous research outside of the bereaved populations, which shows physical activity to be beneficial to mental health [26, 29, 65–67]. Some common grief outcomes were not measured in these studies, for example alcohol consumption or self-harm. As such, the focus on grief outcomes is quite narrow, and important impacts of bereavement are understudied. This review calls for more high-quality research in the area of physical activity and bereavement, particularly given the current surge of international bereavement since the start of the COVID-19 pandemic.

The type of physical activity most likely to benefit grief outcomes could not be easily determined, as there were so many types presented in the research. However, they can be summarised under those linked with the outdoors (e.g. walking, running, hiking, canoeing, cycling), activities around relaxation and a focused mind (e.g. yoga, Dan-Jeon, Qigong, mixed martial arts) or team sports (e.g. football, rugby). Most of these were evaluated using qualitative methods, and there was a lack of quality randomised controlled trials evaluating these activities as interventions compared to a comparator group. These studies show that physical activity to support bereavement is promising, yet more rigorous, experimental research is needed. Many studies failed to define type of physical activity, which can impact replication for future interventions. Future work should consider which type of physical activity is most beneficial, whether there are differences between individual as compared to team or group activity, how physical activity impacts upon grief outcomes and whether this differs depending on type of bereavement.

Due to heterogeneity in study design, types of death encountered, physical activities performed and grief outcomes reported, the synthesis of the data extracted from this review was limited to a narrative approach. Many of the studies had low sample sizes, and three studies used the same cohorts of participants or intervention [42, 44, 45, 49, 53, 54]. Considering that 616,014 deaths were registered within the UK in 2018 [1–3], it is important that future research draws from larger populations with better quality research methods, to provide a wider generalisation of results. Only a small number of studies (*n* = 7) that met the inclusion criteria for this review used an experimental/intervention design; therefore evidence from studies with an appropriate control group is limited, making it difficult to draw concrete conclusions. We know the impact of grief is substantial. For example, scholars have noted physical health changes following bereavement such as higher likelihood of hospital visits and longer stays in hospital [68], or impacts on productivity and functioning at work [69]. The need for appropriate interventions is increasing in importance as populations are living longer and we know the age at which a bereavement is experienced can impact the understanding of death and experience of grief outcomes [5, 9, 70]. The review examined participants from aged 6 to 91 years old; however, no paper distinguished the differences between age groups and their different reactions or understandings about death. Those experiencing bereavement in older age may perhaps have other stressors to contend with at the time of bereavement and their needs may be different to those of a younger age [71]. The types of physical activity identified in the review are inconsistent, with both team sports and individual sports identified; therefore, no affirmative conclusions could be drawn about the type of physical activity and the support it may provide to grief outcomes. Additionally, a number of studies failed to provide details on physical activity in terms of what activity was performed, the duration, intensity and frequency, thus limiting the conclusions we can reach in this review. We present the aggregate of all ages in this review, but the difference between grief outcomes and abilities or suitability of different types of physical activity at different ages should be explored in future work.

This review also attempted to describe physical activity interventions that aim to support bereavement, using BCTs. However, only five studies were able to be coded. BCT coding allows for better replication of an intervention or programme of change, and future researchers should make every effort to specify the content of their designs and/or findings using such reporting practices. Conclusions drawn from the use of the MMAT quality assessment tool should also be taken with caution. Whilst half of the studies were deemed to reach 100% in relation to quality as measured by this tool, there were clearly issues in relation to small sample sizes, study design, and potential replicability of findings.

## Conclusion

This review was the first to systematically investigate the role physical activity plays in the lives of those who have been bereaved. By allowing broad search terms, this review was able to evaluate all types of bereavement, with any age, sex, relationship, and grief outcome. Yet even with such a wide lens, only 25 studies met the inclusion criteria. Our review of these studies suggests that physical activity may be one approach to consider for future intervention, with some evidence of its ability to provide benefit to individuals who have experienced bereavement, often having a positive impact on grief outcomes related to mental health. This often occurs in outdoor activities, those with a team nature or those that enable mindfulness and relaxation. However, physical activity levels have been found to decrease directly following a bereavement when there is no structured physical activity or intervention available. To improve grief outcomes and the impact of bereavement, more research is needed into which physical activity interventions are currently available for individuals who have experienced bereavement, how physical activity can support those who have been bereaved, which type of physical activity is used most by the population, which grief outcomes are most improved by physical activity and whether this differs by age, sex, type of death, relationship to the deceased, length of time since death and type of physical activity. Given the recent scale of international death due to the COVID-19 pandemic, there is an urgent need for wider research is in this area. In conclusion, evidence here suggests that physical activity may be a beneficial behaviour for those who have experienced a bereavement and should be considered as a priority for future research and for future interventions.

## Data Availability

Data extraction tables are available upon request.

## References

[1] National Records of Scotland Web (2019). Monthly data on births and deaths registered in Scotland | National Records of Scotland.

[2] NISRA (2019). Suicide statistics | Northern Ireland Statistics and Research Agency.

[3] Office for National Statistics (2019). Deaths registered in England and Wales—Office for National Statistics.

[4] Green EJ, Connolly ME (2009). Jungian family sandplay with bereaved children: implications for play therapists. Int J Play Ther.

[5] Palmer M, Saviet M, Tourish J (2016). Understanding and supporting grieving adolescents and young adults. Pediatr Nurs.

[6] Pitman A, Rantell K, Marston L, King M, Osborn D (2017). Perceived stigma of sudden bereavement as a risk factor for suicidal thoughts and suicide attempt: Analysis of British cross-sectional survey data on 3387 young bereaved adults. Int J Environ Res Public Health.

[7] Brewer J, Sparkes AC (2011). Parentally bereaved children and posttraumatic growth: insights from an ethnographic study of a UK childhood bereavement service. Soc Sci Med.

[8] Crunk AE, Burke LA, Robinson EH (2017). Complicated grief: an evolving theoretical landscape. J Couns Dev.

[9] Dowdney L (2008). Children bereaved by parent or sibling death. Psychiatry.

[10] LaFreniere L, Cain A (2015). Parentally bereaved children and adolescents. J Death Dying.

[11] Dowdney L (2000). Annotation : Childhood bereavement following parental death. J Child Psychol Psychiat Assoc Child Psychol Psychiatry.

[12] Clute MA, Kobayashi R (2013). Are children’s grief camps effective?. J Soc Work End Life Palliat Care.

[13] Nader K, Salloum A (2011). Complicated grief reactions in children and adolescents. J Child Adolesc Trauma.

[14] Aldrich H, Kallivayalil D (2016). Traumatic grief after homicide. Illness Cris Loss.

[15] Anderson H (2010). Common grief, complex grieving. Pastor Psychol.

[16] Armour M (2003). Meaning making in the aftermath of homicide. Death Stud.

[17] Shear MK, Simon N, Wall M, Zisook S, Neimeyer R, Mancini AD (2011). Complicated gried and related beraement issues for DSM-5. Depress Anxiety.

[18] Feigelman W, Jordan JR, Gorman BS (2009). How they died, time since loss, and bereavement outcomes. J Death Dying.

[19] Prigerson HG, Horowitz MJ, Jacobs SC, Parkes CM, Aslan M, Goodkin K (2009). Prolonged grief disorder: psychometric validation of criteria proposed for DSM-V and ICD-11. PLoS Med.

[20] Agerbo E, Nordentoft M, Mortensen PB (2002). Familial, psychiatric, and socioeconomic risk factors for suicide in young people: nested case-control study. BMJ..

[21] Hua P, Bugeja L, Maple M (2019). A systematic review on the relationship between childhood exposure to external cause parental death, including suicide, on subsequent suicidal behaviour. J Affect Disord.

[22] Dimeo F, Bauer M, Varahram I, Proest G, Halter U (2001). Benefits from aerobic exercise in patients with major depression : a pilot study. Br J Sports Med.

[23] Dunn AL, Trivedi MH, Kampert JB, Clark CG, Chambliss HO (2005). Exercise treatment for depression: Efficacy and dose response. Am J Prev Med.

[24] Elliott C, Sliwa K, Burton R (2014). Pregnancy and cardiac disease. S Afr Med J.

[25] Dadvand P, Bartoll X, Basagaña X, Dalmau-Bueno A, Martinez D, Ambros A, Cirach M, Triguero-Mas M, Gascon M, Borrell C, Nieuwenhuijsen MJ (2016). Green spaces and general health: roles of mental health status, social support, and physical activity. Environ Int.

[26] McDowell CP, MacDonncha C, Herring M (2017). Brief report: associations of physical activity with anxiety and depression symptoms and status among adolescents. J Adolesc.

[27] McMahon EM, Corcoran P, O’Regan G, Keeley H, Cannon M, Carli V (2017). Physical activity in European adolescents and associations with anxiety, depression and well-being. Eur Child Adolesc Psychiatry.

[28] Sarkar M, Fletcher D (2014). Psychological resilience in sport performers: a review of stressors and protective factors. J Sports Sci.

[29] Shachar K, Ronen-Rosenbaum T, Rosenbaum M, Orkibi H, Hamama L (2016). Reducing child aggression through sports intervention: the role of self-control skills and emotions. Child Youth Serv Rev.

[30] Sato M, Jordan JS, Funk DC (2016). A distance-running event and life satisfaction: the mediating roles of involvement. Sport Manag Rev.

[31] Rosenbaum S, Vancampfort D, Steel Z, Newby J, Ward PB, Stubbs B (2015). Physical activity in the treatment of Post-traumatic stress disorder: a systematic review and meta-analysis. Psychiatry Res.

[32] Clough P, Houge Mackenzie S, Mallabon L, Brymer E (2016). Adventurous physical activity environments: a mainstream intervention for mental health. Sports Med.

[33] Centre for Reviews and Dissemination (2009). Systematic Reviews: CRD’s guidance for undertaking reviews in health care.

[34] Mansoubi M, Pearson N, Clemes SA, Biddle SJ, Bodicoat DH, Tolfrey K, et al. Energy expenditure during common sitting and standing tasks: examining the 1.5 MET definition of sedentary behaviour. BMC Public Health. 2010;15 Available from: https://www.ncbi.nlm.nih.gov/pmc/articles/PMC4448542/pdf/12889_2015_Article_1851.pdf. Cited 2018 Jul 23. 10.1186/s12889-015-1851-x PMC4448542 26021449

[35] Mendeley (2018). Mendeley.

[36] Hoffmann TC, Glasziou PP, Boutron I, Milne R, Perera R, Moher D (2014). Better reporting of interventions: template for intervention description and replication (TIDieR) checklist and guide. BMJ.

[37] Michie S, Stralen MMV, West R (2011). Implementation Science The behaviour change wheel: a new method for characterising and designing behaviour change interventions Michie et al. The behaviour change wheel: A new method for characterising and designing behaviour change interventions. Implement.

[38] Pluye P, Robert E, Cargo M, Bartlett G. Proposal: a mixed methods appraisal tool for systematic mixed studies reviews. Montréal McGill Univ. 2011;(1):1–8 Available from: http://mixedmethodsappraisaltoolpublic.pbworks.com/w/file/84371689/MMAT2011criteriaandtutorial2011-06-29updated2014.08.21.pdf. Accessed 2 May 2019.

[39] Stahl ST, Smagula SF, Dew MA, Schulz R, Albert SM, Reynolds CF (2020). Digital monitoring of sleep, meals, and physical activity for reducing depression in older spousally-bereaved adults: a pilot randomized controlled trial. Am J Geriatr Psychiatry.

[40] Huberty J, Sullivan M, Green J, Kurka J, Leiferman J, Gold K (2020). Online yoga to reduce post traumatic stress in women who have experienced stillbirth: a randomized control feasibility trial. BMC Complement Med Ther.

[41] Chen, Gill T, Prigerson H (2005). Health behaviors associated with better quality of life for older bereaved persons. J Palliat Med.

[42] Kang HY, Yoo YS (2007). Effects of a bereavement intervention program in middle-aged widows in Korea. Arch Psychiatr Nurs.

[43] Li J, Chan JSM, Chow AYM, Yuen LP, Chan CLW (2015). From body to mind and spirit: qigong exercise for bereaved persons with chronic fatigue syndrome-like illness. Evid Based Complement Alternat Med.

[44] McClatchey IS, Vonk ME, Palardy G (2009). Efficacy of a camp-based intervention for childhood traumatic grief. Res Soc Work Pract.

[45] Yoo YS, Kang HY (2006). Effects of a bereavement intervention program on depression and life satisfaction in middle aged widows in Korea. J Korean Acad Nurs.

[46] Gorman R, Cacciatore J (2020). Care-farming as a catalyst for healthy and sustainable lifestyle choices in those affected by traumatic grief. NJAS - Wageningen J Life Sci.

[47] Huberty JL, Coleman J, Rolfsmeyer K, Wu S. A qualitative study exploring women’s beliefs about physical activity after stillbirth. BMC Pregnancy Childbirth. 2014;14(26) Available from: https://bmcpregnancychildbirth.biomedcentral.com/track/pdf/10.1186/1471-2393-14-26?site=bmcpregnancychildbirth.biomedcentral.com. Cited 2017 Oct 7. 10.1186/1471-2393-14-26 PMC3901770 24433530

[48] Huberty J, Leiferman JA, Gold KJ, Rowedder L, Cacciatore J, McClain DB (2014). Physical activity and depressive symptoms after stillbirth: informing future interventions. BMC Pregnancy Childbirth.

[49] McClatchey IS, Wimmer JS (2012). Healing components of a bereavement camp: children and adolescents give voice to their experiences. J Death Dying.

[50] Richardson VE (2010). Length of caregiving and well-being among older widowers: implications for the dual process model of bereavement. J Death Dying.

[51] Simpson C, Allegra JC, Ezeamama AE, Elkins J, Miles T (2014). The impact of mid- and late-life loss on insomnia: findings from the health and retirement study, 2010 cohort. Fam Commun Health.

[52] Moores TS, Castle KL, Shaw KL, Stockton MR, Bennett M (2007). “Memorable patient deaths”: Reactions of hospital doctors and their need for support. Med Educ.

[53] Brewer J, Sparkes AC. Young people living with parental bereavement: Insights from an ethnographic study of a UK childhood bereavement service. Soc Sci Med. 2011;72(2):283–90 Available from: https://www.sciencedirect.com/science/article/abs/pii/S0277953610007938. Cited 2018 Aug 13. 10.1016/j.socscimed.2010.10.032 21146275

[54] Brewer J, Sparkes AC (2011). The meanings of outdoor physical activity for parentally bereaved young people in the United Kingdom: Insights from an ethnographic study. J Adv Educ Outdoor Learn.

[55] Phoenix C, Orr N (2017). Analysing exceptions within qualitative data: promoting analytical diversity to advance knowledge of ageing and physical activity. Qual Res Sport Exerc Health.

[56] Granek L, Barbera L, Nakash O, Cohen M, Krzyzanowska MK (2017). Experiences of Canadian oncologists with difficult patient deaths and coping strategies used. Curr Oncol.

[57] Granek L, Barrera M, Scheinemann K, Bartels U (2016). Pediatric oncologists’ coping strategies for dealing with patient death. J Psychosoc Oncol.

[58] Granek L, Ariad S, Shapira S, Bar-Sela G, Ben-David M (2016). Barriers and facilitators in coping with patient death in clinical oncology. Support Care Cancer.

[59] Zhao J, Chi P, Li X, Tam CC, Zhao G (2014). Extracurricular interest as a resilience building block for children affected by parental HIV/AIDS. AIDS Care.

[60] Grimby A, Johansson AK, Sundh V, Grimby G (2008). Walking habits in elderly widows. Am J Hosp Palliat Med.

[61] Gyasi RM, Phillips DR (2020). Risk of psychological distress among community-dwelling older adults experiencing spousal loss in Ghana. Gerontologist..

[62] Wicker P, Orlowski J. Coping with adversity: physical activity as a moderator in adaption to bereavement. J Public Health (Oxf). 2020:1–8 Available from: https://search.ebscohost.com/login.aspx?direct=true&db=cmedm&AN=32529255&site=ehost-live&scope=site&custid=s5099118. Accessed 2 May 2019. 10.1093/pubmed/fdaa059 32529255

[63] Zhang B, Mitchell SL, Bambauer KZ, Jones R, Prigerson HG (2008). Depressive symptom trajectories and associated risks among bereaved Alzheimer disease caregivers. Am J Geriatr Psychiatry.

[64] Michie S, Richardson M, Johnston M, Abraham C, Francis J, Hardeman W (2013). The behavior change technique taxonomy (v1) of 93 hierarchically-clustered techniques: building an international consensus for the reporting of behavior change interventions. Ann Behav Med.

[65] Fox KR (1999). The influence of physical activity on mental well-being. Public Health Nutr.

[66] Masters N (2014). Parkrun eases the loneliness of the long-distance runner. Br J Gen Pract.

[67] Stathopoulou G, Powers MB, Berry AC, Smits JAJ, Otto MW (2006). Exercise interventions for mental health: a quantitative and qualitative review. Clin Psychol Sci Pract.

[68] Tseng FM, Petrie D, Wang S, Macduff C, Stephen AI (2018). The impact of spousal bereavement on hospitalisations: evidence from the Scottish Longitudinal Study. Heal Econ (United Kingdom).

[69] Wilson J (2014). Ward staff experiences of patient death in an acute medical setting. Nurs Stand.

[70] Brent DA, Speece MW, Lin C, Dong Q, Yang C (1996). The development of the concept of death among Chinese and U.S. children 3–17 years of age: from binary to “Fuzzy” concepts?. J Death Dying.

[71] Bennett MK, Soulsby LK (2012). Wellbeing in bereavement and widowhood. Cris Loss.

